# Going (Reo)Viral: Factors Promoting Successful Reoviral Oncolytic Infection

**DOI:** 10.3390/v10080421

**Published:** 2018-08-11

**Authors:** Tarryn Bourhill, Yoshinori Mori, Derrick E. Rancourt, Maya Shmulevitz, Randal N. Johnston

**Affiliations:** 1 Department of Biochemistry and Molecular Biology, Cumming School of Medicine, University of Calgary, Calgary, AB T2N 4N1, Canada; tarryn.bourhill@ucalgary.ca (T.B.); rancourt@ucalgary.ca (D.E.R.); 2 Department of Gastroenterology, Nagoya City West Medical Center, Kita-Ku, Nagoya 467-8601, Japan; ysnmori@yahoo.co.jp; 3 Department of Medical Microbiology and Immunology, Li Ka Shing Institute of Virology, University of Alberta, Edmonton, AB T6G 2E1, Canada; shmulevi@ualberta.ca

**Keywords:** oncolytic virus, reovirus, oncolysis, susceptibility

## Abstract

Oncolytic viruses show intriguing potential as cancer therapeutic agents. These viruses are capable of selectively targeting and killing cancerous cells while leaving healthy cells largely unaffected. The use of oncolytic viruses for cancer treatments in selected circumstances has recently been approved by the Food and Drug Administration (FDA) of the US and work is progressing on engineering viral vectors for enhanced selectivity, efficacy and safety. However, a better fundamental understanding of tumour and viral biology is essential for the continued advancement of the oncolytic field. This knowledge will not only help to engineer more potent and effective viruses but may also contribute to the identification of biomarkers that can determine which patients will benefit most from this treatment. A mechanistic understanding of the overlapping activity of viral and standard chemotherapeutics will enable the development of better combinational approaches to improve patient outcomes. In this review, we will examine each of the factors that contribute to productive viral infections in cancerous cells versus healthy cells. Special attention will be paid to reovirus as it is a well-studied virus and the only wild-type virus to have received orphan drug designation by the FDA. Although considerable insight into reoviral biology exists, there remain numerous deficiencies in our understanding of the factors regulating its successful oncolytic infection. Here we will discuss what is known to regulate infection as well as speculate about potential new mechanisms that may enhance successful replication. A joint appreciation of both tumour and viral biology will drive innovation for the next generation of reoviral mediated oncolytic therapy.

## 1. Introduction

### 1.1. Oncolytic Viruses

Oncolytic viruses (OVs) are an emerging addition to the arsenal of cancer therapeutics. These viruses are capable of selectively targeting and destroying cancer cells while leaving healthy cells unharmed. The notion that viruses could be used as a cancer therapeutic was first investigated in the 20th century after anecdotal reports of patients entering transient remissions subsequent to natural viral infections. An example of this type of report was documented by Bierman et al. who described the phenomenon in a 4-year-old boy with lymphatic leukaemia. Following infection with chickenpox, the child went into remission for a period of one month but eventually succumbed to his disease [1]. Following multiple similar case reports, the use of viruses as anti-cancer agents was investigated, but due to the safety concerns and the limited efficacy, research into this field was largely curtailed in the 1970s and 1980s. The advent of genetic engineering along with enhanced understanding of both cancer and viral biology led to a subsequent revival of the field, beginning in the 1990s. Development of viruses that are more potent, specific and safe led to this renewed interest [2]. Many of these novel viruses are currently in clinical trials for cancer treatment. The first virus approved for clinical use was an engineered adenovirus H101 that received regulatory approval from the Chinese government for the treatment of head and neck cancers in 2005. A recent breakthrough in the field was seen in 2015 with the approval by the Food and Drug Administration (FDA) of a modified herpes simplex virus (or talimogene laherparpvec [TVEC], brand name Imlygic) for the treatment of melanoma [3]. Shortly after this, wild-type reovirus (brand name Reolysin) was granted orphan drug status from the FDA and European Medicines Agency (EMA) for the treatment of gastric, pancreatic and other cancers [4,5,6,7].

OVs are a promising alternative to current therapeutics as they have the potential to differentiate between cancerous and healthy tissues, replicating preferentially in the former. This selection is either natural or engineered depending on the particular virus. Viruses can be engineered to target cancerous cells through the exploitation of aberrant signalling that is a hallmark of numerous cancers. An example of this would be the adenovirus E1B 55K deletion mutant, where the adenoviral gene that encodes the E1B viral protein is deleted in this engineered virus. E1B is required for the prevention of apoptosis in infected cells and allows for extended viral replication. The E1B protein binds to and inactivates p53, a tumour suppressor protein that regulates DNA repair and cell cycling. The deletion prevents effective viral replication in healthy cells with functional p53 [8]. However, the deletion of the E1B gene does not prevent adenoviral replication in cancerous cells that have mutated p53, which is a frequently occurring mutation in many cancers. Some viruses, such as reovirus, are inherently able to preferentially target many types of cancerous cells. However, each OV cannot infect and lyse all cancer types, and selective viral tropism is regulated by a complex interplay of host and viral factors. The requirements are unique to each virus as their replication cycles are varied and distinct.

The various OVs have multiple mechanisms of action responsible for the elimination of cancerous cells. This is advantageous as cancers often subvert chemotherapeutic mediated apoptosis by developing resistance, and OVs have been noted to still be capable of killing tumour cells that have developed resistance to standard chemotherapeutics [9]. There are three main mechanisms by which OVs act: (1) direct viral lysis of cancer cells, (2) induction of an immune response to viral as well as tumour specific antigens and (3) vascular breakdown within the tumour microenvironment [10]. Viral directed lysis of cells is a complicated process that is dependent on numerous factors that are described below. Following successful replication and lysis, newly synthesised viral particles can go on to infect neighbouring tumour cells, propagating and enhancing the viral infection. Alternatively, immune-mediated clearance of tumours arises as a secondary consequence of the viral induction of lysis within the tumour cells. This causes the release of various antigens that stimulate immune cells present in the tumour microenvironment. Damage associated molecular patterns (DAMPS), pathogen associated molecular patterns (PAMPs) and tumour specific antigens escape into the tumour microenvironment upon cell lysis [11]. The release of these antigens can stimulate the maturation of antigen presenting cells within the tumour microenvironment and subsequently leads to the expansion of antigen specific CD4^+^ and CD8^+^ T Cells found in local draining lymph nodes [12]. Tumour specific antigens are particularly important as they prime the immune system for the clearance of additional or distant tumour cells that may not necessarily be infected with virus [13]. As a third mechanism of action, viruses such as vaccinia virus and herpes simplex virus (HSV) have been shown to infect the endothelial cells that are part of the tumour vasculature; the disruption of this vasculature is responsible for the indirect death of cells that may not be infected by virus [14,15]. The tumour vasculature is an appealing target for oncolytic viral infection, as destruction of the neovasculature could provide an entry point for systemically administered virus and thereby also help recruit immune cells to the site of lytic death. Reovirus alone does not readily infect endothelial cells within tumour vasculature; however, when cells are treated with an isoform of VEGF-A (VEGG_165_), endothelial cells could sustain transient reoviral infection leading to lysis. Similarly, brief exposure of VEGF inhibitors such as sunitinib to endothelial cells also transiently supports reoviral infection and lysis, enhancing oncolytic efficiency in immunocompetent mice [16]. Although it was originally thought that the direct lysis of cancers cells was the main determinant of OV efficacy, there is growing evidence that immune clearance and tumour vascular damage can also be major components of effective tumour clearance. Thus, debate continues within the community as to which mechanism is the main catalyst for OV efficacy.

### 1.2. Fundamental Biology Directs Viral Engineering and Combination Therapy

The success of any OV therapy likely depends on the complex interplay between the virus and host and will depend on the biological properties of each specific virus and each particular cancer cell type. This insight has enabled the tailored engineering of viruses for an enhanced oncolytic effect. Knowledge of the dysregulated molecular pathways in cancers has allowed for their exploitation for improved selectivity, as is the case with the adenovirus E1B 55K deletion mutant mentioned above. Deletion of virulence factors within certain viral vectors, such as thymidine kinase from the vaccinia virus (VV), enhances the safety profile of these vectors [17]. The use of miRNA target sites within the viral genomes to restrict viral replication to cancerous cells is another example of how understanding the fundamental biology of cancers is helping to enhance the design of oncolytic vectors [18]. This approach exploits the differences in miRNA expression between heathy and cancerous cells, thereby ensuring selective replication. Another example of viral engineering is seen in adenovirus that has been engineered to express matrix-degrading enzymes such as proteinase K, hyaluronidase and DNAse1. These enzymes enhance viral spread through extracellular matrix, ultimately improving viral dissemination throughout the tumour [19]. The next generation of engineered viruses is being armed with suicide genes and immune stimulatory molecules for enhanced therapeutic outcomes. In the case of reovirus, it is a double stranded RNA virus that is difficult to engineer, although a plasmid based reverse genetics system does exist to allow manipulation of the viral genome [20]. This system has been largely used to investigate the role of reoviral gene segments in processes such as apoptosis, pathogenesis, assembly and disassembly. These investigations have led to the identification of regions within the reoviral genome that can accommodate transgenes. One of the first examples of an autonomously replicating genetically engineered reovirus was developed by Hoeben’s group, who incorporated the fluorescent protein iLOV (improved Light, Oxygen or Voltage sensing domain from Arabidopsis thaliana) into the σ1 binding region of reovirus [21]. The reverse genetics plasmid based system is continually being improved, and the use of African swine fever virus NP868R capping enzyme to enhance protein expression and reovirus rescue is a recent example [22]. It is also important to note that many natural reoviral mutants have been isolated that offer alternatives to current reoviral therapy [23,24,25]. None of these mutant (engineered or naturally occurring) reoviral strains has yet progressed to the clinic. A detailed discussion of the numerous engineered strains of reovirus is beyond the scope of this review and we refer the reader to a recent publication by Mohamed et al., for further details [26], plus a paper by Howells et al. [27]*.*

An appreciation for the molecular mechanisms invoked by chemotherapeutics has also aided in the rational design of combination therapies with OVs. The distinct mechanisms utilised by OVs and chemotherapeutics may be complementary and thereby result in synergistic responses for improved patient outcomes. An example of this type of synergism is that of 5-Fluorouracil (5-FU) and adenovirus combination therapy. 5-FU is a pyrimidine antimetabolite and acts by inhibiting thymidylate synthase, which results in the prevention of thymidine (required for DNA replication) production [28]. The combination of 5-FU and adenovirus is thought to be synergistic as 5-FU pre-treatment increases the expression of coxsackie and adenovirus receptor (CAR) [29]. CAR is the main receptor utilised by adenovirus (serotype 5) to gain entry into the cell and is often downregulated in cancers. There is an increase in viral uptake in cells with low CAR expression when they are pre-treated with 5-FU. There have been subsequent efforts to engineer adenovirus to augment 5-FU toxicity with the intention of reducing dosage and overcoming resistance. There are numerous examples of how the exploitation of OV lysis in combination with standard chemotherapeutics has resulted in enhanced effect, and additional details may be found in a recent review by Wennier et al. [28].

### 1.3. Reovirus

Numerous viruses have entered clinical trials to evaluate their potential as oncolytic agents, although many of the questions regarding their core biology remain unresolved. Reovirus is the only wild-type (non-engineered) OV approved for use in the clinic and is one of the most advanced in its study. Reovirus received an orphan drug designation from the FDA for numerous cancers, including ovarian, gastric, pancreatic and malignant glioma, in 2015 [4,5,6,7]. Last year, Reolysin also received fast track designation from the FDA for the treatment of malignant breast cancer. Although reovirus is well-studied, many aspects of its tropism, replication and spread are debated and unknown.

Reovirus is an acronym for Respiratory Enteric Orphan virus and is weakly or non-pathogenic, although it can cause mild gastroenteritis, coughing and various other flu-like symptoms in young children [30,31]. Reovirus has also been associated with neonatal biliary atresia [32]. In immune deficient mice, reovirus has been known to cause “black foot” syndrome, which is the localised haemorrhage or thrombosis in the extremities of mice [33]. Recently, reovirus has also been implicated in the initiation of celiac disease by promoting the loss of tolerance to dietary antigens [34]. Reovirus is a highly stable non-enveloped virus with two concentric protein layers, comprising the outer capsid and the inner core. The outer capsid is a 20 sided icosahedral structure and is roughly 80 nm in diameter. The inner core encases 10 gene segments of double stranded RNA, which comprise the viral genome [35]. There are small, medium and large gene segments, each of which encodes proteins that are named with corresponding Greek letters sigma (σ), mu (μ) and lambda (λ).

Reovirus was originally isolated from the stools of children by Rosen and Sabin in the 1950s and is part of the *Reoviridae* family [30,31]. Other members of this family include rotavirus, which is most commonly responsible for severe paediatric gastroenteritis, and bluetongue virus, which causes disease in ruminants [36,37]. There are four main mammalian orthoreoviral serotypes studied in laboratories (Types 1–4) that have been identified through antibody neutralisation and hemagglutination-inhibition; among these, the strains most commonly used (one for each serotype, respectively) are Lang, Jones, Dearing and Ndelle [30,38].

The wild-type Dearing strain (T3D) is the only reoviral strain that has entered clinical trials for oncolytic therapy. Due to reovirus’ natural tropism for transformed cells and its relatively non-pathogenic profile, it is considered an ideal candidate for oncolytic therapy [39,40]. Reovirus is well-tolerated in clinical trials and a maximum tolerated dose has not been achieved. However, while reoviral monotherapy has been very effective in vitro and in animal studies, its benefits in humans have often been brief and modest with few clinical trials showing desired levels of anticancer efficacy [41]. This is not unique to reovirus, as many OVs have shown disappointing efficacy in the clinic compared to pre-clinical evaluations in animal models. Even so, the low toxicity profile associated with reovirus encourages its use in combination with standard chemotherapeutics, which have led to desirable synergistic responses in clinical trials [41]. There is much debate over the reason for the low efficacy of reoviral monotherapies. A better understanding of reoviral biology could help clarify what is occurring within the clinic. Indeed, reoviral tropism has yet to be thoroughly explained, and we still do not fully appreciate why some cancers are highly susceptible to reoviral oncolysis while others remain unaffected by infection. Discerning the mechanisms responsible for a productive oncolytic infection are vital for developing potent anti-cancer therapeutics.

Reovirus follows general principles of viral replication that include virus attachment and entry across the cellular lipid bilayer, delivery of nucleic acids to an intracellular site, usurpation of cellular factors for viral replication and generation of new viral progeny and finally, induction of cellular lysis and viral release. In this review we will explore the factors that promote successful oncolytic viral infection with a focus on reovirus.

## 2. Delivery

### 2.1. Barriers Preventing Infection

The first step in any productive oncolytic infection is effective delivery to the host cells. A sufficient number of viral particles need to reach cancerous cells before they are cleared by the innate immune system. These viral particles also have to navigate their way through physiological barriers such as hypoxic environments, high interstitial pressure and extracellular matrix (ECM) before they can reach their target cells [10,42]. Many OVs are administered via intratumoural injection, allowing for enhanced viral replication as the virus is delivered directly to a niche that optimally supports replication and lysis. Systemic administration can also be an attractive option for treatment as it has the added advantage of treating both primary and metastatic tumours. However, systemic delivery can be challenging in the case of reovirus, as it is ubiquitous and found in water bodies around the world [26]. The virus predominantly infects children, which can result in an adult population with up to 100% seropositivity [43,44,45]. Thus a majority of the human population has neutralising antibodies (NAB) targeted toward reovirus, which can result in viral clearance even before it reaches the tumours. While many cancer patients are severely immunocompromised due to standard therapeutic regimens (which might be expected to enhance viral activity), a study by White et al. demonstrated that patients that had received prior chemotherapy were nevertheless able to mount effective antibody responses to reoviral infection [46]. This indicates that immune mediated clearance of reovirus may be a significant barrier to effective treatment and is often cited as a reason for reduced efficacy in clinical trials, especially when patients are not screened for their reoviral seropositivity prior to virotherapy.

### 2.2. Circumventing Immune Clearance

Immune clearance is a potential challenge for any OV, not just reovirus, as a majority of the population carries NABs toward measles, adenovirus and other viruses as a result of primary exposure or vaccination [10]. Strategies to circumvent immune surveillance and clearance have therefore been investigated. In viruses such as adenovirus, genetic engineering was used to exchange capsid proteins with a related adenovirus that does not infect humans, a process known as pseudotyping. For viruses that are not easily engineered, as is the case with reovirus, alternative solutions have been investigated. For example, Sakurai et al. demonstrated enhanced tumour killing abilities in reoviral vectors coated with a cationic liposome transfection reagent. However, the benefits of liposome coating were not due to immune evasion, but rather it promoted cellular infection and endosomal escape [47].

An alternative approach to avoid immune clearance of oncolytic viruses is to have them “carried” to tumours in migratory cells, such as dendritic cells. There are many properties a carrier cell must possesses to be an effective delivery vehicle for OV therapy. First, the cell must have a degree of intrinsic tumour homing ability to selectively deliver OVs. Carrier cells also need to act as viral replication factories upon arrival at a tumour site. If viral replication and lysis occurs too rapidly within carrier cells, they will be detected by the immune system and cleared before reaching the tumour. Loading of OVs into a carrier cell is usually performed ex vivo with the objective of packaging as many viral particles in the cell as possible.

In addition to dendritic cells, T cells have been used as carrier cells and in the case of reovirus were shown to be effective at producing sustained cancer clearance as a result of induction of anti-cancer immune response in murine models when compared to mature dendritic cells [48]. Melcher and colleagues have further emphasised the idea of immune cell carriage of reovirus. In a window-of-opportunity clinical trial they demonstrated that reovirus is capable of replicating within tumours after systemic administration despite the presence of NABs. The group postulates that this is because reovirus is capable of evading the NABs by hitchhiking on peripheral blood mononuclear cells (PBMCs) [49]. Although the group previously demonstrated the ability of dendritic and T cell carriage of reovirus when infected ex vivo*,* there is little convincing evidence that systemic administration of the viruses results in similar carriage [48]. An alternate explanation that was not fully explored in the paper was that productive viral infection within tumours might occur first, and the cells identified as transporting reovirus toward the tumour may actually be circulating toward lymph nodes to activate T cell responses. The group did not look at activated T cell responses to viral particles at the same time point as their investigation into cell carriage (it would be of interest to note if an immune response toward the virus had already been stimulated as early as 1 h post infusion). The group also did not provide evidence that the granulocytes and platelets that were identified as carrying reovirus were localised within resected tumours.

Stem cells have also been proposed to have the qualities of an ideal carrier as they are inherently attracted toward tumours, they are immunosuppressive and they can facilitate OV replication. Two types of stem cells have been investigated for their use as carriers for multiple OVs, namely mesenchymal and neuronal stem cells [50]. In this context, reovirus has been shown to infect and lyse embryonic stem cells in vitro and in vivo. These stem cells are sensitive to infection and undergo lysis within 48 h of infection. Reovirus was shown to be capable inducing apoptosis in teratomas in vivo that were initiated by implantation of embryonic stem cells into immunocompromised mice [24]. However, embryonic stem cells would not likely be an ideal vehicle for the transport of reovirus as lysis occurs rapidly within this cell population [24]. Park and Kim investigated reoviral replication in mesenchymal stem cell populations and found that these cells were still capable of proliferation and differentiation following reoviral infection. They concluded that mesenchymal stem cell populations would be a potential carrier for reovirus to enhance systemic infections [51].

## 3. Receptor Attachment and Entry

### 3.1. Receptor Mediated Selectivity

Viral tropism is largely predicated on the expression of specific receptors that facilitate viral attachment and entry into the cell [52]. For example, the attenuated measles virus that is used as an OV and for vaccination has evolved to use CD46 and nectin as specific receptors [53]. The attenuated HSV-1 oncolytic viruses use herpesvirus entry mediator to gain entry into the cell [53,54]. Many of these receptors are overexpressed on cancers, thereby allowing higher infection rates in tumours than in healthy tissues and creating a therapeutic window [42,54]. Adenovirus (Ad5) predominantly uses the CAR receptor to mediate viral attachment and entry. The CAR receptor is rarely expressed in tumours and so adenovirus is often engineered to express differing capsid and fibre proteins (pseudotyping), facilitating entry through receptors such as CD46 that are abundant in tumours [55]. Many viruses such as Newcastle disease virus or vaccinia virus enter a cell through mechanisms such as endocytosis, and their precise receptors are presently unknown [42].

Reovirus has specific attachment and entry receptors, but their expression is not necessarily well correlated with viral tropism [52]. Reovirus can attach to cells through low affinity interactions between the viral spike protein sigma 1 (σ1) and the ubiquitous sialic acid [56,57]. There are differences among reovirus strains in sialic acid usage: type 1 Lang interacts with ganglioside GM2 glycan and strain 3 Dearing interacts with a linked 5-*N*-acetylneuraminic acid (Neu5Ac) [36,58,59]. It is not fully understood how reovirus gains access to its primary high affinity receptor, junction adhesion molecule 1 (JAM-1), which is found in tight junctions [60]. Reoviral infection does not appear to damage tight junction integrity [61]. Terasawa et al. demonstrated that JAM-1 expression did not correlate with differential susceptibility to reovirus induced cell death in 19 different cancer cell lines. This demonstrates that the expression of reoviral receptors may be necessary for viral entry, but is not necessarily a limiting factor leading to the selective killing of tumour cells [52].

### 3.2. Viral Entry and Uncoating

Reovirus enters the cell through clathrin-mediated endocytosis, which is facilitated by the attachment of lambda 2 (λ2, the turret protein at the base of the spike) to beta 1 (β-1)-integrins [62]. Vesicles that contain the internalised reovirus are then transported via microtubules to late endosomes. Reovirus is then exposed to acidic pH and cathepsin protease degradation, which results in viral uncoating (loss of σ3 capsid protein, cleavage of μ1 protein to form δ and ϕ and conformational changes in the σ1 protein) [59,63,64,65]. The intermediate virion that forms as a result of this process is known as an infectious subviral particle (ISVP). Following this, viral cores are released from the lysosome into the cytoplasm where viral replication begins to take place [59]. Alain et al. demonstrated that variable uncoating and capsid degradation could be a mechanism determining reoviral selectivity, as ISVPs prepared by exposure of intact virus to chymotrypsin could bypass barriers to cytoplasmic entry and were capable of infecting untransformed NIH3T3 cell lines [66,67,68] that are normally highly resistant to the virus. Dauzenberg and colleagues further demonstrated that this uncoating could also affect reoviral selectivity in cancerous cell lines. U-118 MG, a glioblastoma cell line that is normally resistant to infection and that does not express JAM-A, was grown in 3D spheroid cultures. This group determined that exposure to proteases cathepsin B and L could modify reoviral particles, allowing oncolytic infection to occur in the resistant cell line independent of JAM-A expression [23]. In this context it is interesting to note that reovirus may also be exposed to proteases within the extracellular tumour microenvironment, which may have implications for specificity in patients.

The natural niche for mammalian reovirus is cells within the gastrointestinal tract. Viral particles are exposed to proteases trypsin and chymotrypsin after ingestion and before they encounter their host cells. These proteases degrade the outer capsid of reovirus and result in the formation of ISVPs [47,69]. It is interesting that the ISVPs and intact virions undergo different trafficking upon internalisation. ISVPs appear to bypass the endocytic pathway at an early stage [70]. Another recent study seems to indicate the presence of microbiota within the intestinal tract may play a key role in mediating Reoviral infection, where mice treated with antibiotics and displaying reduced intestinal microbiota also had decreased reoviral infection and pathology [71]. This has implications for the role of sialic acids in mediating reoviral tropism. It is known that reoviral mutants that have reduced sialic acid binding do not spread efficiently to secondary sites of infection (brain, spleen and liver) [72]. There are other spontaneous mutants that rely mainly on sialic acid binding for infection. These are known as Jin (JAM-A independent) mutants and were isolated from U-118MG glioblastoma cells. Alterations in the sialic acid binding pocket in the shaft of the spike protein (σ1) modified viral tropism and allowed infection in a variety of resistant cell lines [73]. Although sialic acid displays a low affinity interaction with reovirus, it can determine tropism to some degree. Even so, as sialic acid is overexpressed in many cancers, it still does not fully explain the variations seen in reoviral susceptibility [74].

It was initially speculated that the reoviral preference for certain transformed cells was mediated through high levels of epidermal growth factor receptor expression (EGFR). Murine cell lines were transfected with genes encoding EGFR and the susceptibility of these cells to reoviral lysis was increased [75]. Later it was shown that cells transfected with the *v-erbB* oncogene also had increased susceptibility to reovirus infection. The *v-erbB* oncogene encodes a truncated version of EGFR, lacking the extracellular domain but with an active tyrosine kinase domain. This led to the revised idea that activation of intracellular signalling cascades was instead responsible for reoviral selectivity, and specifically the upregulation of the *ras* oncogene signaling pathway [76], although a specific mechanism by *which ras* pathway activation may lead to enhanced viral replication and cytolysis remains elusive.

## 4. Viral Replication and Appropriation of Host Intracellular Machinery

As viruses are obligate intracellular molecular parasites, they all rely on their host to provide factors that are required for viral replication. There is a continuous battle waged between the host cell and the virus, resulting in an arms race between the two. Host cells have evolved many novel mechanisms to detect and destroy viral invaders, while the viruses evolve to circumvent detection and usurp control of cellular signalling for enhanced viral replication. Accordingly, there are numerous intracellular mechanisms at work within a cell that either act to help or hinder viral replication.

### 4.1. Intracellular Immune Surveillance

**Figure 1:**
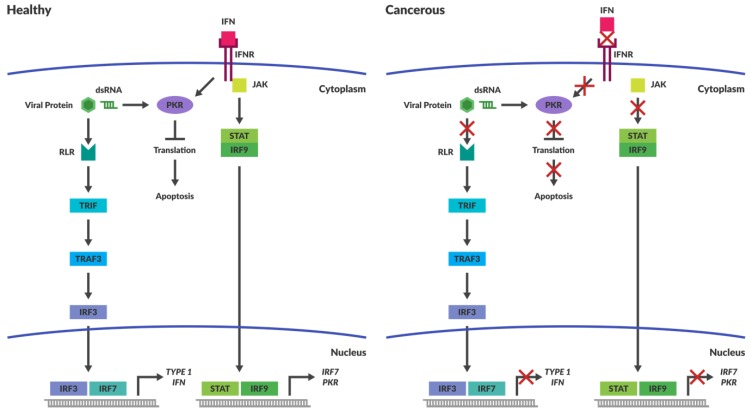
Intracellular immune Responses in Cancerous and Healthy Cells. The schematic depicts intracellular immune responses stimulated by either interferon or the detection of intracellular pathogens in healthy and cancerous cells. In healthy cells the detection of pathogens results in the activation of pathways that stimulate apoptosis. Cancerous cells are less responsive to induction of antiviral responses caused by the production of cytokines, such as Interferon (IFN). Intracellular molecules responsible for pathogen detection are often non-functional within cancers and prevent activation of apoptotic pathways in the presence of viral pathogens. Abbreviations: IFN—Interferon; IFNR—Interferon Receptor; JAK—Janus Kinase; STAT—Signal Transducer and Activator of Transcription; IRF—Interferon Regulated Factor; PKR—Protein Kinase dsRNA-regulated.

In healthy cells, interferon signalling is one of the main mechanisms by which viral invasion is inhibited. Interferon release enables cells to warn their neighbours of viral infection, which safeguards against viral spread. Interferon signalling activates numerous downstream pathways that result in cellular apoptosis. Some of the downstream factors that are involved with oncolytic viral clearance include interferon regulatory factors (IRF) 3, 7 and 9 as well as tumour necrosis factor-associated factor 3 (TRAF3), retinoic acid-inducible gene 1 (RIG-I) and other RIG-like receptors (RLRs). One of the major pathways stimulated by interferon type 1 binding to its receptors is the JAK-STAT (Janus kinase–signal transducer and activator of transcription) pathway [42].Activation of this pathway augments IRF 7 expression and subsequent interferon production, as well as the activation of anti-viral machinery. IRF 3 and 7 are the transcription factors responsible for type 1 interferon expression while IRF 9 binds STAT1 and 2 to drive transcription of interferon stimulated genes such as IRF7 and protein kinase R (PKR) (Figure 1).

**Figure 2:**
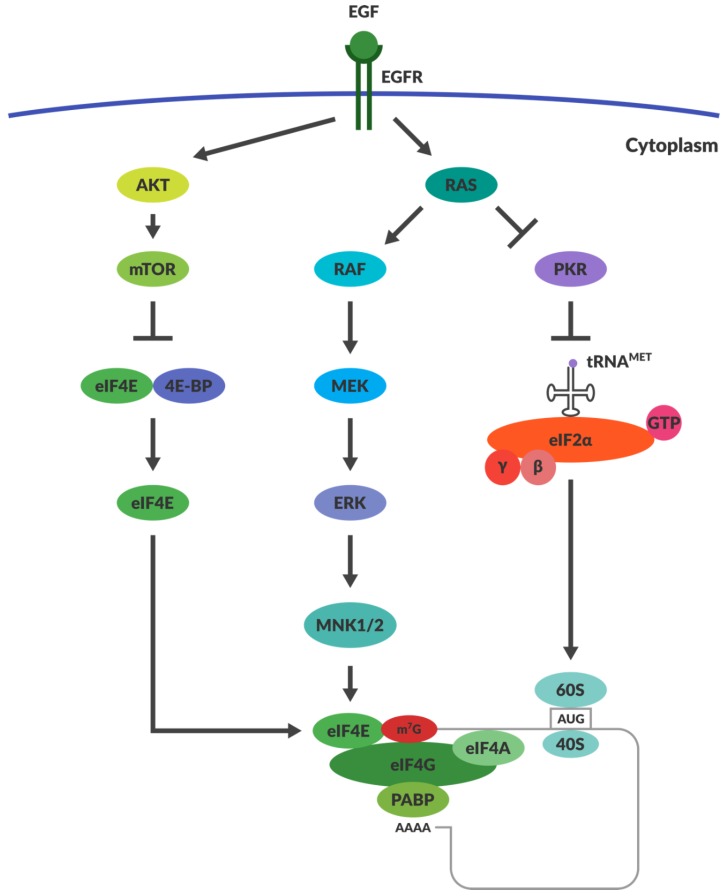
Regulation of Translation within Cancers. The mechanisms responsible for translational regulation are illustrated in the diagram. Overexpression of RAS drives inhibition of PKR, thereby allowing the function of eIF2α. RAS activation stimulates the activation of MNK1/2, which facilitates eIF4E function. AKT overexpression prevents the function of inhibitor 4E-BP. This allows eIF4E to function as a cap binding protein within the ternary complex formed during translation. Abbreviations: EGF—Epithelial Growth Factor; EGFR—Epithelial Growth Factor Receptor; PKR—Protein Kinase dsRNA-regulated; eIF2α—eukaryotic Initiation Factor 2α; eIF4G—eukaryotic Initiation Factor 4G; eIF4E—eukaryotic Initiation Factor 4E; eiF4A—eukaryotic Initiation Factor 4A; PABP—PolyA binding protein; AKT—Protein kinase B; mTOR—mechanistic target of rapamycin; 4E-BP—4E Binding Protein.

PKR has the innate capacity to recognise ds RNA and once activated, it inhibits protein synthesis throughout the cell (Figure 1), achieved through its phosphorylation of eukaryotic initiation factor 2α (eIF2α) [77]. Phosphorylation of eIF2α increases its affinity for its binding partner eIF2B, thus preventing eIF2α from recycling back to its GTP-bound form. eIF2α and GTP–eIF2 (containing α, β and λ subunits) function together as a ternary complex to recruit the initiator methionyl tRNA during translational initiation (Figure 2) [78]. Termination of protein synthesis causes rapid apoptosis and viral clearance, followed by activation of immune cells and stimulation of further innate immune responses [79].

To circumvent immune recognition, viruses produce their own proteins that counteract the activity of PKR. The σ3 protein of Reovirus is responsible for inhibiting PKR and thus allowing viral translation to continue during infection [80]. Recently, reoviral protein μNS was shown to sequester IRF3 and prevent its nuclear translocation, thereby preventing subsequent IFN stimulation [81]. Differences among reoviral strains in IFN stimulation have also been linked to differential IRF3 activation [81], and the reoviral µ2 protein has also been shown to block IFN signalling through the nuclear accumulation of IRF9 [82]. In HSV, infected cell protein 34.5 (ICP34.5) and unique short 11 glycoprotein (US11) are the proteins responsible for hindering innate immunity recognition. ICP34.5 activates protein phosphatase-1A, which dephosphorylates eIF2α and permits continued viral replication [83,84]. US11 directly inhibits the activation of PKR, providing a secondary mechanism to maintain protein translation [85]. The oncolytic version of HSV virus has deletions in the genes that encode both these proteins, thereby targeting these viruses toward the unique intracellular niche in tumours where PKR activation is defective.

### 4.2. Cancers Have Compromised Immune Responses

It is well established that many tumours have an immune suppressive microenvironment to avoid immune recognition and the subsequent destruction of mutated cells. The process by which tumours evolve to evade the immune system is known as immunoediting and is beyond the scope of this review, but readers are referred to an excellent review by Schreiber et al. [86]. A result of this immunoediting is that the tumour cells are less responsive to induction of antiviral responses caused by the production of self-protective cytokines such as IFN-1 and-2 or tumour necrosis factor (TNF) [10]. Often the intracellular pathways responsible for pathogen detection are compromised within cancers (Figure 1). This provides viruses with an ideal niche for replication and lysis.

### 4.3. OVs Take Advantage of the Aberrant Signalling in Cancers

Viruses such as herpes simplex and adenovirus have been engineered to take advantage of the aberrant signalling within the tumour cells that would otherwise prevent their replication in normal healthy tissues [42]. Often virulence factor genes are removed from the OV to make them more safe and specific. In the case of HSV, deletion of the ICP34.5 and US11 genes results in the virus being detected and blocked within healthy cells, but it is replication competent in defective cancer cells [42,87,88]. RAS upregulation has also been implicated in enhancing reoviral spread by suppressing virally induced production of IFN-β. RAS transformed cells are severely comprised in their ability to both produce and respond to IFN-β. This has been attributed to the negative regulation of RIG-I signalling which prevents viral recognition [68]. In the context of a tumour cell that is mutationally active in the RAS pathway, PKR activity is also suppressed; several oncolytic viruses take advantage of this defect in PKR signalling, such as vaccinia virus [89]. Thus it has been proposed that RAS over-activation and consequent PKR inactivation might in part be responsible for reoviral tropism and selection seen in Ras-active cancers [90].

### 4.4. Does RAS Activation and PKR Inactivation Determine Reoviral Susceptibility?

After it was shown that cells transfected with the *v-erbB* oncogene have increased susceptibility to reoviral infection, it was thought that downstream intrinsic pathways were responsible for reoviral selectivity. The main downstream molecule investigated was RAS, which is a primary target of EGFR stimulation. EGFR stimulation results in autophosphorylation of the receptor’s cytoplasmic domains. This recruits phosphotyrosine-binding adaptor molecules such as Shc and Grb2. Grb2 attracts son of sevenless (SOS) protein, which functions as a guanine nucleotide exchange factor that is responsible for the activation of RAS by the exchange of RAS-GDP with RAS-GTP [91,92]. NIH3T3 cells were transfected with either RAS or SOS oncogenes and this greatly enhanced their susceptibility to reoviral infection [90]. The inhibition of viral translation appears to be an important bottleneck for reoviral replication in untransformed cells. Reoviral attachment, entry and viral transcription were similar in transformed and untransformed cells [93]. It was previously shown that PKR could influence the selectivity of reoviral replication and lysis [80]. Strong et al. then used a PKR specific inhibitor within non-transformed cells that resulted in the restoration of viral protein translation and reoviral replication [90]. The exact mechanism coordinating RAS transformation and PKR mediated reoviral selectivity has yet to be elucidated. Indeed, RAS activation has also been implicated in enhancing numerous aspects of reoviral infection such as virion disassembly during entry, viral progeny production and virus release [67,68]. While RAS downregulation of PKR remains an important possible explanation for enhanced reovirus replication within certain cancers (Figure 2), there is nevertheless much debate within the field as to whether RAS and PKR are indeed the limiting factors in reoviral tropism.

### 4.5. RAS Activation May Not Be Responsible for Reoviral Tropism

Contrary to the model presented above, numerous groups have shown that the simple activation of RAS and inactivation of PKR do not fully explain reoviral selectivity, causing ongoing debate within the field as to the mechanisms that truly govern reoviral tropism. Sakurai’s group demonstrated that RAS activation did not fully correlate with reoviral killing in multiple transformed cell lines. They showed that some cell lines with low levels of RAS activity were highly susceptible to reoviral lysis while cell lines with high levels of RAS activity could be resistant to infection. The group went on to demonstrate that cathepsin L and B expression were suitable biomarkers for reoviral susceptibility [52]. Smakman et al. demonstrated that knockdown of RAS did not affect reoviral infection or replication in colorectal cancer cell line that have the Kras^D12^ mutation (KRAS is constitutively active due to this mutation) [94]. They went on to demonstrate that reovirus was capable of replicating within a cell line that did not have functional KRAS, where the gene was deleted through homologous recombination. The cell line was an isogenic derivative of the KRAS^D12^ lines used in their original studies [95]. Work done by van Holt and colleagues demonstrated that reovirus was incapable of infecting cell cultures or tissue fragments taken from colorectal tumours irrespective of the presence of activated KRAS [96].

Zhang et al. went on to further show that PKR was not a critical determinant for reoviral tropism. This group used siRNA mediated knockdown of PKR expression in tumour cells and showed it did not lead to elevated levels of reoviral lysis [97]. Similarly, Twigger et al. found that inhibiting PKR activity in resistant cell lines did not bring sensitivity to reoviral infection. Furthermore, inhibition of RAS activation did not prevent reovirus replication in squamous cell carcinomas of the head and neck [98]. Schiff’s group analysed reoviral infection in cells lacking PKR and RNAse L, and found that the presence of RNAse L and PKR slightly enhanced reovirus replication, contrary to the RAS-PKR model described above [99]. The Schiff group also found that reovirus replicated more efficiently in the presence of active eIF2α, and that replication was decreased in PKR knock-out murine embryonic fibroblasts (MEF). The degree to which viral replication was affected was strain specific but all strains followed a similar trend. The group also noted that phosphorylation of eIF2α had two interesting effects: firstly, its activation resulted in an increase in the expression of ATF4 (a transcription factor that helps cells recover from stress) and secondly, that eIF2α phosphorylation led to the formation of stress granules. The authors hypothesised that the formation of these stress granules gave reovirus a competitive advantage for translation [100]. Reoviral transcripts are placed in close proximity to limited translation factors when stress granule formation is induced [100]. Overall, these studies suggest that the original RAS activation and PKR inactivation model does not completely explain reoviral selection, despite initial hopes and the positive early results. The model also does not adequately explain why reovirus is capable of infecting and killing healthy embryonic stem cell populations. There are also many cancerous cell lines that do not conform to the predicted selection criteria. Thus the factors that ultimately promote successful oncolytic infection by reovirus remain elusive and this has led to studies of alternative mechanisms of control.

### 4.6. Alternative Proposed Intracellular Mechanisms Governing Reoviral Tropism

Regulation of cellular and reoviral translation are promising avenues of investigation. Once reoviral core particles are released from late stage endosomes, conformational changes in the core capsid proteins and loss of σ1 (the spike protein) enable reoviral RNA transcription [65,101,102,103]. The viral cores contain the necessary machinery to begin replication of the dsRNA genome, namely RNA-dependent RNA polymerase (λ3), guanylyltransferase and methyltransferase (λ2) [59,91]. Primary transcripts are capped and released into the cytoplasm where cellular ribosomes are responsible for the production of new viral proteins. Positive-sense RNA and new viral proteins assemble into core particles. DsRNA is then synthesised within the core particle. Non-structural proteins µNS, σNS and structural protein μ2 promote the formation of viral factories where viral replication and viral assembly take place [26,59].

As noted above, stress granule formation has been suggested as a promoter of productive reoviral infection, as these granules have high concentrations of translational initiation factors and 40S ribosome subunits. Stress granules are formed within cells when there are high levels of stress that prevent or stall translation. They are thought to be temporary repositories for stalled 43S and 48S ribosomal preinitiation complexes. Once stress conditions have abated, cellular gene expression can rapidly resume as these complexes are released into the cytoplasm [104]. Interestingly, kinases (such as PKR) that have been known to phosphorylate eIF2α can contribute to stress granule formation. Viruses can block or co-opt stress granule formation to promote their own replication. Unlike most viruses, reovirus induces the formation of stress granules independently of PKR activity [105,106]. The stress granules are disassembled once viral protein translation begins, which may enhance the preferential translation of viral proteins even in the presence of phosphorylated eIF2α [105].

Viruses can also use other mechanisms to ensure the translation of viral proteins, such as regulating capped and uncapped translation. Some in the field believe that early reoviral replication is dependent on capped mRNA and progresses toward the use of uncapped transcripts later in infection. Surprisingly, uncapped reoviral transcripts are translated with higher efficiency than their capped counterparts. It is thought that reoviral protein σ3 is responsible for the translational efficiency of the uncapped late viral mRNA, but the mechanism by which σ3 accomplishes this is unknown [37,107,108]. Recently it has been shown that σNS also plays a role in RNA binding during viral replication [109]. Other viruses that impair host translation of capped mRNAs operate by preventing the phosphorylation of the initiation factor 4E-BP1 [110]. When 4E-BP1 is not phosphorylated, it binds to and inactivates eIF4E, which is responsible for binding the cap on mRNA transcripts (Figure 2) [110,111]. It is unlikely that 4E-BP1 regulates reoviral tropism, however, as cancer cells often express high levels of mTOR, which phosphorylates 4E-BP1 and promotes capped translation [79]. RAS overexpression may also lead to the prevalence of capped translation over uncapped. ERK, a downstream kinase substrate of the RAS pathway, can upregulate the expression of MAPK interacting kinases (MNK1-3). MNKs assist the binding of eIF4E to eIF4G, which ultimately facilitates capped translation (Figure 2) [78]. However, inhibitors of MNK1 (CGP57380 and cercosporamide) had no effect on reoviral cytolysis and replication (unpublished data). If reoviral replication can occur independently of RAS-RAF-MEK-ERK and PI3K pathway activation, then it would be unlikely that MNK1 kinase or 4E-BP would be critical in the enhancement of reoviral translation [93].

Another aspect to translational control includes regulation by post-transcriptional mechanisms present within the cell, such as RNA interference. Regulation of mRNA expression (both viral and cellular) can be modulated by microRNAs and other non-coding RNA transcripts. MiRNAs are small non-coding RNA segments of about 20 nucleotides in length and are incorporated into a protein complex known as the RNA-induced silencing complex (RISC) within the cytoplasm. The miRNAs have complementary sequences toward their target mRNAs and upon binding to these sequences the RISC complex prevents mRNA translation either through mRNA degradation or translational repression [112]. Thus as may be expected, viruses have many interesting interactions with miRNA that are currently under investigation [110]. Enterovirus 71 stimulates the expression of miRNA-141, which prevents the expression of eIFG and in turn shuts-off host translation [113]. Hepatitis C virus has a unique interaction with miRNA-122. Binding of the miRNA to the viral ssRNA genome enhances viral translation by enhancing ribosomal recruitment [114]. Intriguingly, HSV encodes its own miRNAs that can regulate both cellular and viral mRNA translation [115]. It has also been argued that reovirus can downregulate miRNA-Let-7 expression, which promotes caspase 3 expression and enhances apoptosis [116]. OVs that are easy to engineer, such as measles virus and vaccinia virus, have been engineered to encode miRNAs or to encode target sites for particular miRNAs [18,117]. The target sites take advantage of differential miRNA expression between healthy and tumourigenic cells. In healthy cells where the miRNAs are abundantly expressed, viral replication is prevented. However, tumours with dysfunctional miRNA production will not be able to prevent viral replication, thereby improving viral selectivity [27,110].

In addition to short non-coding RNAs, a recent study has implicated long non-coding RNAs (lncRNA) in the regulation of viral replication. The lncRNA regulates cellular metabolism through activation of the metabolic enzyme glutamic-oxaloacetic transaminase 2 (GOT2) [118,119]. This work shows that viruses can usurp cellular metabolism through regulation of long non-coding RNA. It remains to be seen if this highly conserved regulatory mechanism is harnessed by reovirus.

## 5. Apoptosis Induction

### Reoviral Mediated Apoptosis

For OVs to be effective, they need to induce cell death or immune targeting of the tumour cell that they infect. Each OV uses specific mechanisms to induce varying kinds of cell death. The extent to which reovirus induces cell death is strain specific, with T3D inducing apoptosis to a greater extent than T1L [120]. Proteins that appear to be required for the initiation of reovirus induced apoptosis include σ1, σ1s and μ1 [92,121]. σ1 is responsible for binding sialic acid and JAM-1, thus viral attachment is necessary to promote replication and subsequent apoptosis. However, receptor binding alone has not proved to be sufficient to cause apoptosis. The role of σ1s has been difficult to determine as it appears to be dispensable for apoptosis in cultured cells, although the severity of the apoptotic response in mice is greater when σ1s is present. It is thought that σ1s influences the kinetics of apoptosis induction and enhances viremia and viral dissemination in vivo [122,123,124]. The activation of apoptotic pathways occurs after viral disassembly but before cores become transcriptionally active within the cytoplasm. This implicates a role for the μ1 protein which has a role in membrane penetration and endosomal escape [125,126]. It is proposed that μ1 induces pro-apoptotic signalling once viral particles have escaped the endosome [121].

Reovirus is known to induce apoptotic cell death through both extrinsic and intrinsic pathways. Infection causes the expression of TNF-related apoptosis-inducing ligand (TRAIL), a ligand that initiates the extrinsic apoptotic pathway (death receptor pathway). TRAIL binds to its death receptor (DR-5) and this mediates interactions with the Fas-associated death domain (FADD). Ultimately, this results in caspase-8 activation followed by caspase-3 and -7 stimulation, which facilitates cell death [89,127,128]. Reovirus can also induce intrinsic apoptosis where cytochrome c and second mitochondrion-derived activator of caspase (Smac/DIABLO) are released from the mitochondria, resulting in caspase stimulation and subsequent cell death [24,129,130].

Numerous intracellular pathways have been implicated in reovirus induced apoptosis. Reovirus is known to stimulate the NF-κB pathway, which is essential for activating pro-apoptotic proteins such as Noxa independently of IFN-β induction. It is interesting that reoviral activation of the NF-κB pathway takes much longer than if the pathway were stimulated by TNF, indicating the possibility a viral agonist is responsible for its stimulation [131,132]. Mitogen activated kinases have also been shown to mediate apoptotic effects. Specifically, the c-Jun NH2-terminal kinase (JNK) pathway appears crucial for apoptosis. Inhibitors of this pathway do not interfere with viral replication but prevent apoptotic death. An active c-Jun protein is not a requirement for apoptosis, meaning the JNK pathway may use a different mechanism to initiate cell death [59,133]. Recently, work published by Lee’s group has demonstrated a novel role for RAS in JNK-mediated apoptosis [134]. They demonstrated that reoviral infection sequestered RAS to the Golgi body by decreasing RAS palmitoylation. This prevents its migration to the cell membrane and prevents its correct function. Sequestration of RAS to the Golgi body causes the stimulation of MEKK1/MKK4/JNK signalling that ultimately results in cellular apoptosis [134].

Reoviral infection also stimulates an immune response in healthy animals. We have discussed the action of PKR at length, however it is not the only molecule responsible for detection of intracellular pathogens (Figure 1). Toll-like receptors are surface and intracellular molecules that are responsible for the detection of pathogens. Once PAMPs are recognised, innate immune stimulation occurs and antiviral mechanisms are activated. Pattern recognition molecules such as retinoic acid-inducible gene I (RIG-I) and melanoma differentiation-associated protein 5 (MDA-5) are responsible for detecting dsRNA within the cytoplasm [135,136]. These molecules activate Interferon Response Factor 3 (IRF3) leading to interferon production (Figure 1) [42]. Reovirally induced apoptosis is dependent upon IRF3 stimulation but independent of interferon-β production [131,135]. Recently it has been shown that Reovirus is capable of inducing necroptosis, mediated through a two step activation process. Initially incoming genomic viral RNA is detected via cytoplasmic sensors (RIG-I/MDA-5) thus initiating IFN production. The second step required for induction of necroptosis was determined to be synthesis of new genomic dsRNA [137].

## 6. Effects of the Tumour Microenvironment

Once lysis of an infected cell has occurred, OVs are secreted into the tumour microenvironment where they are faced with barriers to dissemination. The microenvironment outside of a host cell is an important factor that determines viral spread and efficacy. The immune cells and cytokines present in the tumour microenvironment are of particular importance to the OV mediated anti-cancer effect.

Induction of cell death has the effect of stimulating an immune response to viral particles and initiating the second mechanism by which OVs clear cancerous cells. Different viruses will induce different types of cell death. Some are more immune-stimulatory (necrosis and pyroptosis) than others (apoptosis) [42]. In the last decade, there has been a paradigm shift in the way OV mediated responses are perceived. In light of work done with regards to tumour immunology, many OVs are thought to act primarily as stimulants that reactivate suppressed immune cells within the tumour microenvironment. It is now proposed that immune activation of cytotoxic T cells is perhaps the dominant mechanism for OV mediated tumour clearance [69].

### 6.1. Development of Cancer Immunotherapies

Tumours have evolved a number of mechanisms to escape immune detection [86]. The production of cytokines that suppress the immune response such as interleukins-6, IL-10 and transforming growth factor-β prevent dendritic and antigen presenting cells from functioning correctly [138]. This immune suppressive microenvironment promotes the generation of T regulatory responses [138]. Tumours can also prevent immune cell infiltration via their negative effects on vascularisation and T cell adhesion. Tumours can furthermore prevent the expression of T cell attractive chemokines such as CCL2, 3, 4, 5, 9 and 10, which may hinder trafficking of T cells to tumour beds [10,139]. Tumour cells can also avoid immune detection through intracellular mechanisms, such as preventing the expression of major histocompatibility complex proteins [140].

One of the most important mechanisms of immune evasion involves commandeering immune checkpoints. Immune checkpoint pathways are used to dampen immune response and prevent chronic immune stimulation. The two major checkpoints that have received a lot of attention include cytotoxic T-lymphocyte-associated protein 4 (CTLA-4) and Programmed Cell Death protein 1 (PD1) [141]. Tumour cells often express PD-L1, which is the ligand for PD-1, and their interaction prevents T cell effector function. CTLA-4 works in a slightly different manner. It is expressed on T cells and competes for binding to the B7 molecule expressed on many cancerous cells. Once CTLA-4 is bound to B7 their interaction prevents T cell activation within lymphoid organs [141]. The characterisation of these interactions has resulted in the development of monoclonal antibodies directed toward CTLA-4 (ipilimumab) and PD-1 (nivolumab and pembrolizumab). These antibodies effectively function to block immune suppression in the tumour microenvironment. These antibody treatments have received regulatory approval from the FDA and have become part of the standard of care for many cancers. Importantly, this knowledge of tumour immunology has altered our perception of OVs, in that many but not all OVs have demonstrated the capacity to change the immune suppressive tumour microenvironment and promote anti-tumour immunity.

### 6.2. Debate Around the Importance of OV Immune Stimulation

Recently, work presented by Li et al. demonstrated that T cell responses were the major contributors to adenovirus-induced tumour clearance in an immune competent Syrian hamster model [142]. This group developed antibodies directed toward CD3^+^ positive T cell populations, thereby effectively preventing T cell function. They showed that deletion of T cell function impaired adenovirus’ ability to mediate tumour clearance. Long term tumour recurrence was also elevated in the absence of T cell response. Elevated and durable levels of infectious viral particles within the tumour were also detected when T cells were impaired, but this did not correlate to enhanced anti-tumour efficacy. Cyclophosphamide was then utilised to cause depletion of total white cell count, which enhanced viral lysis and anti-tumour efficacy. The authors argue this demonstrates the importance of T cell mediated responses to oncolytic infection and tumour clearance [142]. They also show that hamsters pre-immunised against adenovirus could effectively eradicate adenoviral infected tumour cells but this had little to no effect on anti-tumour efficacy. Viral re-administration effectively enhanced therapeutic outcome in this model [142]. This paper provides a convincing argument for the importance of immune mediated anti-tumour effects when using adenovirus.

In a similar study conducted by Hirasawa et al., an immunocompetent mouse model was used to determine the effect immune clearance had on intravenously delivered reovirus [143]. Similar to Li et al., this group used antibodies directed toward CD4^+^ and CD8^+^ T cells as well as cyclophosphamide to abrogate immune responses. In this instance, however, reoviral lysis and anti-tumour efficacy were enhanced when immune cells were inactivated. Not only was there a reduction in tumour size in mice that were immunosuppressed and treated with reovirus, but enhanced survival was also noted [143]. Mice pre-immunised against reovirus had a reduction in the anti-cancer efficiency, nonetheless this was restored upon immune suppression. Reoviral administration with immune suppression prevented reoccurrence of primary tumours [143]. This group suggested that the disruption of the antiviral host immune responses was the reason for enhanced oncolytic effect [143]. It is clear that these two papers express conflicting results, however this may simply be a consequence of the particular OV used. The predominant mechanisms that are responsible for anti-tumour efficacy are likely to be virus specific. In the case of reovirus, Melcher’s group has shown that immune mediated clearance of tumour cells is important for therapeutic effect. In their work the group demonstrate that immune clearance can be mediated upon introduction of Reovirus through T cell carriage. This clearance occurs in the absence of reoviral replication. It is unsurprising that reovirus is capable of stimulating the immune system—it is, after all, a foreign antigen and pathogen. Melcher’s group did not compare effective tumour clearance between cancers that display viral replication and oncolysis vs. those that do not permit reoviral replication [144]. It is difficult to know which mechanism contributes the most to the overall effectiveness of reoviral therapy. It is clear however that at least both mechanisms contribute the potency of reoviral tumour clearance. Since the development of immune checkpoint inhibitors, research into mechanisms of primary reoviral oncolysis has become less common, whereas studies of the role of the immune system in reoviral oncolysis and combination therapies with these checkpoint inhibitors are increasingly seen [145,146,147,148,149,150,151,152]. It is even so clear based on the evidence provided by Hirasawa and colleagues that understanding the precise mechanisms that regulate direct oncolytic lysis will nevertheless be of critical importance for the optimal development of this oncolytic therapy.

### 6.3. Tumour Microenvironment and Reoviral Dissemination

The tumour microenvironment has many other influences on reoviral replication. It is important to remember that reovirus naturally targets cells within the gastrointestinal tract. The niche in which reovirus has evolved is harsh. Before viral particles encounter host cells, they are exposed to proteases such as chymotrypsin and trypsin, resulting in the formation of ISVPs that may enter susceptible cells more readily [153,154]. The tumour microenvironment is also known to overexpress particular proteases and this may further aid reoviral dissemination. This may be an important factor when determining reoviral susceptibility in vivo. For example, it has been shown previously that U118 cell lines are not susceptible to reoviral infection in vitro*,* yet in vivo U118 derived tumours regressed upon intratumoural injection [66]. This suggests that the tumour microenvironment may support reoviral infection when cells in culture do not. This highlights the impact that the microenvironment has on OV selectivity.

Another example of niche factors that impact viral replication both by affecting intracellular signalling and extracellular spread is the amount of oxygen present in the microenvironment. The natural niche for reoviral infection (intestinal and respiratory tracts) is arguably exposed to higher levels of oxygenation than would be present within the tumour microenvironment, and indeed hypoxic conditions within tumours are commonly observed. It has been shown that hypoxic conditions can negatively affect adenoviral and VSV replication but this does not appear to be the case for reovirus [155,156,157].

## 7. Discussion

Since its isolation almost 60 years ago, a great deal has been learned about reovirus, from its structural and genomic components to its innate tropism for certain transformed cells. Despite the immense body of knowledge regarding this virus, there remain important gaps in our fundamental understanding of its biology. In particular, we still do not fully comprehend viral tropism for susceptible cancer cells. Despite much effort, there are still no biomarkers that indicate unambiguously whether or not productive infection will take place. As we have discussed throughout this article, viral selectivity is mediated by both viral as well as host factors, yet there remain many unanswered questions regarding the factors which are most important for governing productive reoviral infection.

A major challenge to the use of reovirus as an OV is immune clearance, as the majority of the adult population has NABs towards the virus. This has prompted investigations into alternative methods for systemic delivery, one of the most interesting being the use of stem cells as delivery agents. These investigations have yielded confounding results such as the fact that embryonic stem cells are sensitive to reoviral infection while adult mesenchymal stem cells appear to be resistant. It will be of critical importance to determine the host or viral factors governing the selection between these similar cell types to ensure the safety and efficacy of stem cell vehicles for reoviral transport. It will be interesting to determine the cellular mechanisms that are similar between embryonic stem cells and transformed cells that allow for successful infections to occur. This may provide valuable insights into the mechanisms that govern reoviral tropism.

It is clear that reoviral receptors play a role in determining reoviral sensitivity, even though JAM-1 and sialic acid are both widely expressed and cannot fully account for reoviral tropism. The expression of receptors has been at best only weakly correlated with reoviral susceptibility. The expression of markers such as cathepsin B and L have also been correlated with reoviral susceptibility. Consequently, attachment and viral entry are determined by the presence of cellular factors and can perhaps partly account for reoviral tropism.

Upon entering the cell, host factors play an important role in determining whether infection will lead to productive replication. Although the overexpression of RAS and subsequent inactivation of PKR are often credited with reoviral selectivity, this is not entirely true. There is abundant evidence to demonstrate that RAS expression does not fully correlate with reoviral susceptibility.

The point at which reoviral replication is hindered within non-permissive cells has been determined to be most likely at the level of translation and many other host factors may be involved in the regulation of susceptibility. Yet few studies have focused on the translational regulation of reovirus replication. There are numerous alternative mechanisms that may explain reoviral selectivity in cancerous versus healthy tissue. It will be very interesting to determine if capped versus uncapped translation is a preferred mechanism governing reoviral replication within cancerous and healthy cells. This may provide clues as to the translational machinery that facilitates replication. It is also possible that intracellular factors such as non-coding RNAs may regulate reoviral translation, and it is clear that there are abundant differences between healthy and transformed cells in their non-coding RNA expression. It will be fascinating to determine the role of unconventional non-coding RNA such as lncRNAs in reoviral regulation and whether they are indeed involved in this process. Another exciting avenue of research may involve protein localisation as many viruses are responsible for reorganising the protein distribution within their hosts, perhaps thereby promoting replication efficiency. It is tempting to speculate the reovirus is capable of usurping and localising translation initiation complexes in stress granules that eventually form viral factories. Although a great deal is known about the mechanisms that result in reoviral induced apoptosis, there are still some areas worth further investigation. It is striking that new mechanisms for apoptosis initiation have been brought forward by Lee’s group. The role of RAS in Golgi body fragmentation demonstrates novel mechanisms for well-studied proteins.

The tumour microenvironment has also proved to be an essential component for determining the efficacy of oncolytic infection, particularly in the case of immune clearance. However, the view that OVs are simply an immune adjuvant is misleading, as the contribution to OV efficacy is clearly virus specific. While some viruses have superior immune stimulatory characteristics, many may not fall into this category. Reovirus is one such virus where direct viral lysis of tumour cells appears to be the major contributor to anti-cancer effects. This makes understanding viral tropism a particularly important avenue of discovery as it has consequences for its clinical application.

Finally, reovirus is a wild type virus that causes minimal disease in healthy individuals, yet can mediate direct cell lysis in cancer, even though we still know little about what makes a particular cancer cell susceptible to lysis. Once this is understood, reovirus can perhaps be implemented with better utility within the clinic. Biomarkers can be used to screen patients before they receive treatment to determine if their particular cancer will response to reoviral treatment. Understanding the pathways responsible for reoviral lysis will also allow for the selection of rational therapeutic combinations that are likely to synergise and enhance therapeutic outcome. A full appreciation for reoviral tropism may also allow for the development of molecules that specifically enhance the replication cycle or modify viral tropism. Establishing the mechanisms responsible for a productive oncolytic infection is vital for developing potent, selective and safe anti-cancer therapeutics. A fundamental understanding of tumour and viral biology will help drive innovation for the next generation of reoviral mediated oncolytic therapies.
